# Safety and pharmacodynamic dose response of short-term prednisone in healthy adult subjects: a dose ranging, randomized, placebo-controlled, crossover study

**DOI:** 10.1186/s12891-016-1135-3

**Published:** 2016-07-16

**Authors:** Dona L. Fleishaker, Arnab Mukherjee, Fredrick S. Whaley, Shanthini Daniel, Bernhardt G. Zeiher

**Affiliations:** Pfizer Inc, Eastern Point Rd, Groton, CT 06340 USA; Innovative Analytics, 161 East Michigan Ave, Kalamazoo, MI 49007 USA; Jasper Clinic Inc, 526 Jasper Street, Kalamazoo, MI USA; Astellas Pharma Global Development, Northbrook, IL 60062 USA

**Keywords:** Biomarker, Dose–response, Glucocorticoid, Healthy-subject, Pharmacodynamic, Prednisone, Safety

## Abstract

**Background:**

Glucocorticoids (GCs), such as prednisone, are the standard of care for several inflammatory and immunologically mediated diseases, but their chronic systemic administration is severely limited by serious adverse effects that are both dose and time dependent. Short-term treatment (7–14 days) with oral prednisone is used for many acute inflammatory and allergic conditions. This study was conducted to characterize the safety and pharmacodynamic (PD) dose–response of a 7-day course of oral prednisone on biomarkers of GC receptor agonism.

**Methods:**

In this randomized, single-blind, placebo-controlled, crossover study (A9001309), 37 healthy subjects received placebo or a prednisone dose from 2.5–60 mg daily over 7 days in each of three treatment periods. White blood cell counts and plasma samples for measuring cortisol, osteocalcin and procollagen type 1 N-propeptide (P1NP) were obtained at 2, 4, 8, and 12 h post-dose on Day 1, immediately prior to dosing on Days 1, 2, and 4, and at nominal dosing time on Days 0 and 8. Urine samples for urinary N-terminal cross-linked telopeptide of type 1 collagen (uNTX) were collected on Days 0, 1, 2, 4, and 8. Serum samples for adiponectin were obtained prior to dosing on days 0, 1, 4 and 8.

**Results:**

Daily doses of prednisone up to 60 mg resulted in dose- and time-dependent decreases in plasma osteocalcin, plasma P1NP, serum cortisol, and absolute blood eosinophil counts. Absolute blood neutrophil counts increased, while blood lymphocyte counts rebounded to an increased level following an initial rapid decrease after dosing. An increase was observed for uNTX and adiponectin. The incidence of adverse effects with prednisone was not dose related, and nervous system disorders, mainly headache, were the most frequently reported adverse effects.

**Conclusions:**

This characterization provides important and relevant information on safety and PD responses of short-term prednisone dosing over the commonly-used clinical dose range, and also provides a reference for early clinical development of dissociated agents targeting a differentiated PD profile.

**Trial registration number:**

NCT02767089 (retrospectively registered: 21 April 2016).

## Background

Glucocorticoids (GCs) are commonly used to manage inflammatory and immunologically-mediated conditions [1–3], and continue to have a prominent place in the clinic despite having a profile of serious adverse effects that are dose- and time-dependent [4, 5]. Due to these known serious adverse effects, a GC such as prednisone is used at the lowest effective dose (5–7.5 mg daily) for chronic conditions such as rheumatoid arthritis (RA); the use of higher doses is limited to the shortest treatment duration required for management of acute conditions and disease exacerbations [6, 7]. One of the most prevalent adverse effects is that on bone remodeling, specifically, an uncoupling of bone formation and resorption in favor of bone loss via direct effects on osteoblasts [8]. Indeed, the most common form of iatrogenic osteoporosis is GC induced [9]. Many other adverse effects, such as electrolyte imbalance, weight gain, and metabolic disturbances, result from GC-induced effects on other tissues including the hypothalamic-pituitary-adrenal (HPA) axis [10, 11]. Similarly, due to a plethora of effects on leukocytes and vascular endothelial cells, such as altered cell distribution patterns, immobilization, and apoptosis, GC therapy can result in dramatic changes in circulating white blood cell profiles that may contribute to an increased risk of GC-associated infection [12–14].

Recent drug discovery and development efforts have focused on approaches to reduce adverse effects, while maintaining efficacy of GC therapy. These approaches include development of a modified-release prednisone formulation and discovery of selective GC receptor ligands that putatively dissociate anti-inflammatory effects mediated by genomic transrepression from adverse effects mediated by genomic transactivation [7, 15–18]. Despite the present understanding of the known adverse effects of GC therapy, and recent drug development efforts to potentially dissociate efficacy and safety of GCs, the dose–response and time course of the effect of current GCs on various biomarkers of GC receptor agonism (anti-inflammatory and adverse effects) have not been systematically characterized. The characterization of the safety and pharmacodynamics (PD) of multiple doses of a standard GC such as prednisone, over the commonly used clinical dose range (2.5**–**60 mg once daily), provides important and relevant information for clinical use, as well as reference for early clinical development of dissociated agents targeting a differentiated PD profile.

The present study was conducted to further characterize the safety and dose–response of 7-day prednisone administration using biomarkers of GC receptor agonism in a healthy adult population.

## Methods

### Subjects

Eligible subjects were healthy adult volunteers aged 18–55 years (male) or 18**–**44 years (female), with a body mass index of 18–30 kg/m^2^ and a total body weight >50 kg (110 lb). Subjects with evidence or history of clinically significant hematologic, renal, endocrine, pulmonary, gastrointestinal, cardiovascular, hepatic, psychiatric, neurologic, or allergic disease (including drug allergies, but excluding untreated, asymptomatic seasonal allergies at time of dosing) or any condition possibly affecting drug absorption were excluded from the study.

### Study design

This randomized, single-blind, placebo-controlled, crossover study (A9001309) was designed to characterize the dose–response of prednisone on biomarkers of GC receptor agonism. Within 28 days of screening, all eligible subjects were randomly assigned to one of seven treatment sequences, each with three 7-day treatment periods separated by a 14-day washout period (Table 1). The treatments in each sequence included either three of the six prednisone doses evaluated in the study (2.5, 5, 10, 20, 40, or 60 mg), or two prednisone doses and placebo. In the first treatment period only, all subjects had baseline assessments on Day 0, the day prior to dosing.

**Table 1:** Treatment sequences

Treatment sequence	Subjects, *n*	Treatment period		
		1	2	3
A	5	Placebo	2.5 mg	10 mg
B	5	2.5 mg	5 mg	20 mg
C	5	5 mg	10 mg	40 mg
D	5	10 mg	20 mg	60 mg
E	5	20 mg	40 mg	Placebo
F	5	40 mg	60 mg	2.5 mg
G	5	60 mg	Placebo	5 mg
Total	35^a^			

Doses shown correspond to the daily prednisone dose administered for 7 days in each treatment period

^a^Two subjects discontinued the study during Period 2 and were replaced following approval by the study statistician

### Biomarker evaluations and analytic methods

#### HPA axis

Serum samples for morning cortisol were obtained immediately prior to dosing or nominal dosing time on Day 0 (baseline, day prior to first dosing) and on Days 1 (first day of dosing), 2, 4, and 8, in each of the three 7-day treatment periods. Serum samples for cortisol were obtained at 2, 4, 8, and 12 h following the first sample on Day 0 (Period 1 only) and following the first prednisone dose on Day 1. A radioimmunoassay (Roche Diagnostics, Indianapolis, IN) was used initially for measurement of cortisol in serum, but the results indicated the possibility of assay interference from prednisone and its metabolite prednisolone. Consequently, a specific, validated, high-performance liquid chromatography assay (calibration range, 10–3000 ng/mL) with tandem mass spectrometry (SGS Cephac, Poitiers, France) was used to assay cortisol in plasma samples collected for osteocalcin (OC) measurement on Day 0 and Day 1 (pre-dose and 2, 4, 8, and 12 h post-dose) and prior to dosing on Day 2. These plasma samples were stored at −70 °C until assayed for cortisol and had previously undergone one freeze-thaw cycle. Stability of cortisol was confirmed in plasma for a time period greater than the duration of storage with up to three freeze-thaw cycles.

Serum was also obtained for assaying cortisol levels on Day 8 before and 30 min after low-dose adrenocorticotropic hormone (ACTH) stimulation. Subjects with an abnormal low-dose ACTH stimulation response on Day 8 were administered the test again after 2 weeks. A radioimmunoassay was used for measurement of serum cortisol from the low-dose ACTH stimulation test. Assay interference from prednisone and prednisolone was considered unlikely, since complete washout of both moieties was expected at the time these samples were obtained.

#### White blood cell counts

Complete blood count with differential (data for neutrophils, eosinophils, and lymphocytes are shown) was obtained at 2, 4, 8, and 12 h post-dose on Day 1, immediately prior to dosing on Days 1, 2, and 4, and at nominal dosing time on Days 0 and 8.

#### Bone metabolism

Plasma samples for OC, a biomarker of bone formation, were collected serially on Day 0 (at nominal dosing time and 2, 4, 8, and 12 h thereafter) and serially post-dose on Day 1 (2, 4, 8, and 12 h), immediately prior to dosing on Days 1, 2, and 4, and at nominal dosing time on Day 8; plasma samples for procollagen type 1 N-propeptide (P1NP), also a bone formation marker, were collected 12 h post-dose on Day 1, immediately prior to dosing on Days 2 and 4, and on Days 0 and 8. Urine samples for urinary N-terminal cross-linked telopeptide of type 1 collagen (uNTX), a biomarker of bone resorption, were collected from the second pre-noon voiding of the bladder on Days 0, 1, 2, 4, and 8. OC and uNTX were assayed using an enzyme-linked immunosorbent assay method. P1NP was assayed by a validated radioimmunoassay. A kinetic modification of the Jaffe reaction was used for the quantitative measurement of urinary creatinine (uCr). Pacific Biometrics, Inc. (Seattle, WA, USA) kits were used for all four analytes.

#### Carbohydrate and metabolic effects

Serum samples for fasting glucose and insulin were obtained immediately prior to dosing on Days 0, 1, 2, 4, 6, and 7. For the oral glucose tolerance test (OGTT), the subjects were to ingest 75 g of a glucose solution within 5 min of receiving study medication on Day 6; this solution was to be ingested within 10 min, and blood samples for glucose were then collected at 0.5, 1, and 2 h. Serum samples for triglycerides were obtained immediately prior to dosing on Days 0, 1 and 4, and on Day 8 and, for adiponectin, immediately prior to dosing on Days 0, 1 and 4, and on Day 8. Serum samples were analyzed for adiponectin using a LINCO Diagnostics Services (St. Charles, MO, USA) radioimmunoassay; the validated range of the assay was 2–100 ng/mL.

#### Central nervous system

Subjects were required to complete Profile of Mood State (POMS™) and Medical Outcomes Study: Sleep Scale (MOS-Sleep) questionnaires on the evening of Days 0 and 7.

#### Safety

Adverse events (AEs) were monitored throughout, and vital signs (sitting blood pressure and pulse rate) were performed at screening and prior to dosing on Days 0, 1, 4, and 8; laboratory safety tests (hematology, blood chemistry, urinalysis, and hormone and chronic infection tests), were performed at screening and on Day 0; a post-void weight was taken at screening and on Days 1 and 8 of each treatment period.

### Statistical analyses

The change from baseline in primary biomarker endpoints (biomarkers of AEs and biomarkers of anti-inflammatory activity) for each prednisone dose was compared with the change from baseline for placebo, using a repeated-measures crossover analysis of covariance model containing effects for sequence, period, time, dose, time by dose interaction, and subject within sequence (as random effect), as well as baseline as a covariate. For comparison with placebo, the least squares mean difference, standard error, 95 % confidence interval, and *P* value were reported.

## Results

### Subjects

Overall, 37 subjects were screened; all were assigned to study treatment. Five subjects were assigned to each of the seven treatment sequences (A-G) and received either three active doses of prednisone 2.5, 5, 10, 20, 40, or 60 mg, or two active doses and placebo. Ultimately, each of the treatments was received by 15 or 16 subjects. The proportion of subjects completing the study was 91.9 %. Two subjects in treatment sequence E (prednisone 20 mg, 40 mg, and placebo in Periods 1, 2, and 3, respectively) and one subject in treatment sequence G (prednisone 60 mg, placebo, and prednisone 5 mg in periods 1, 2, and 3, respectively) discontinued from the study. The subject in treatment sequence G discontinued during Period 2 while receiving placebo due to AEs related to the study treatment. The other two subjects discontinued from the study for reasons not related to study treatment; both subjects withdrew consent. One subject in treatment sequence E was also discontinued during Period 2 while receiving prednisone 40 mg; both subjects that discontinued during Period 2 were replaced following approval by the study statistician. The other subject in treatment sequence E was in treatment period 1 at discontinuation, and was not replaced.

### Baseline characteristics

Demographic characteristics were similar among the treatment groups. Subjects were aged between 18 and 50 years, and the majority were white and male (Table 2).

**Table 2:** Demographic characteristics of all treatment groups

Characteristic	Male	Female	Total
	(*n* = 30)	(*n* = 7)	(*N* = 37)
Age, years			
Mean (SD)	33.7 (9.8)	35.6 (6.7)	34.1 (9.2)
Range	18–50	27–43	18–50
Race, *n*			
White	21	5	26
Black	7	1	8
Other	2	1	3
Weight, kg			
Mean (SD)	81.3 (10.2)	74.4 (9.2)	80.0 (10.3)
Range	57.2–101.6	60.8–84.8	57.2–101.6
Height, cm			
Mean (SD)	177.0 (6.7)	160.6 (4.7)	173.9 (9.1)
Range	164.0–188.0	153.7–167.0	153.7–188.0

*SD* standard deviation

### Effect of prednisone on markers of safety and efficacy

#### HPA axis

**Fig. 1:**
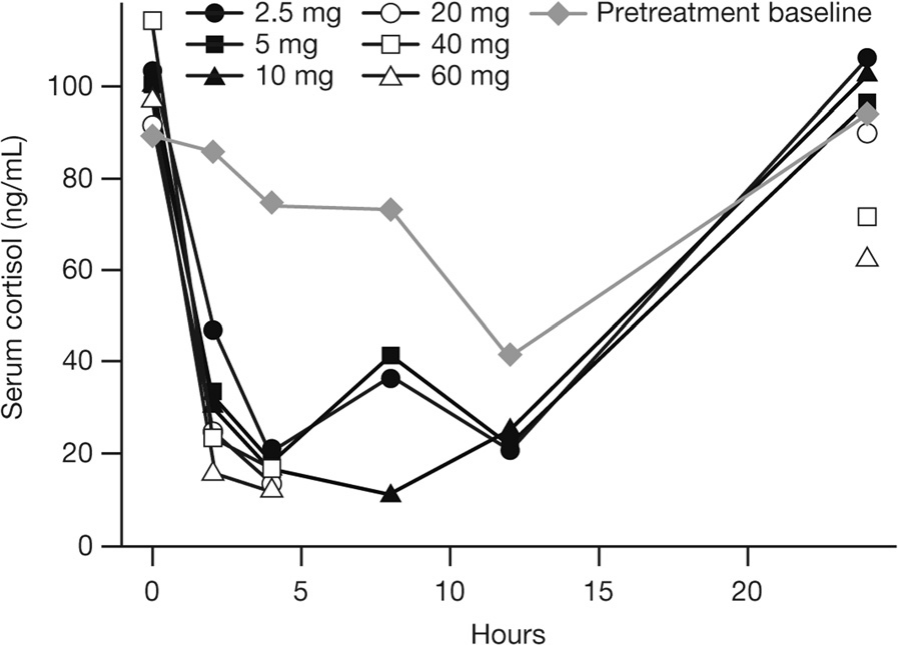
Mean serum cortisol concentrations up to 24 h following the first daily dose of prednisone. Pretreatment cortisol concentrations over 24 h were measured in all subjects the day prior to the first day of dosing in Period 1

Plasma cortisol concentrations decreased rapidly following the first dose of prednisone, and then recovered in a dose-dependent manner (Fig. 1). Following single prednisone doses of 20 mg and higher, all individual cortisol measurements at 8 and 12 h post-dose were below the lower limit of quantification (10 ng/mL), and mean pre-dose cortisol concentrations in these dose groups on Day 2 were lower than the corresponding median values on Day 1 (baseline).

A low-dose ACTH stimulation test was carried out after completion of all treatment periods to assess whether each subject’s HPA axis was adversely affected by prednisone. A normal response to ACTH was defined as an increase in serum cortisol to >18 μg/dL within 30 min of ACTH injection. At the end of treatment, 19/37 subjects demonstrated normal responses to ACTH, and 18 subjects had abnormal responses (serum cortisol outside of normal limits) (Table 3). The 18 subjects returned in 2 weeks, and after a second low-dose stimulation test, 16/18 subjects had normal responses. Two subjects required a third test and one subject required a fourth test before their responses returned to normal. The two subjects who did not achieve a normal response within 2 weeks received prednisone doses of either 40 mg or 60 mg in the last treatment period.

**Table 3:** Number of subjects with abnormal and normal responses to ACTH stimulation tests performed every 2 weeks

Treatment sequence	Subjects, *n*	Period			Subjects with abnormal response to ACTH stimulation, *n*		
		1	2	3	Test 1	Test 2^a^	Test 3^b^
					(*N* = 37)	(*N* = 18)	(*N* = 2)
A	5	Placebo	2.5 mg	10 mg	3	0	0
B	5	2.5 mg	5 mg	20 mg	5	0	0
C	5	5 mg	10 mg	40 mg	4	1	1^c^
D	5	10 mg	20 mg	60 mg	5	1	0
E	6^d^	20 mg	40 mg	Placebo	0	0	0
F	5	40 mg	60 mg	2.5 mg	0	0	0
G	6^d^	60 mg	Placebo	5 mg	1	0	0
Total	37				18	2	1

Doses shown correspond to the daily prednisone dose administered for 7 days in each treatment period

*ACTH* adrenocorticotropic hormone

^a^Subjects who failed Test 1 were re-tested 2 weeks later

^b^Subjects who failed Test 2 were re-tested 2 weeks later

^c^Returned to normal on Study Day 138

^d^One subject in sequence E and one in sequence G discontinued the study during Period 2 and were replaced following approval by the study statistician, therefore *n* = 6 in these groups for this analysis

#### White blood cell counts

**Fig. 2:**
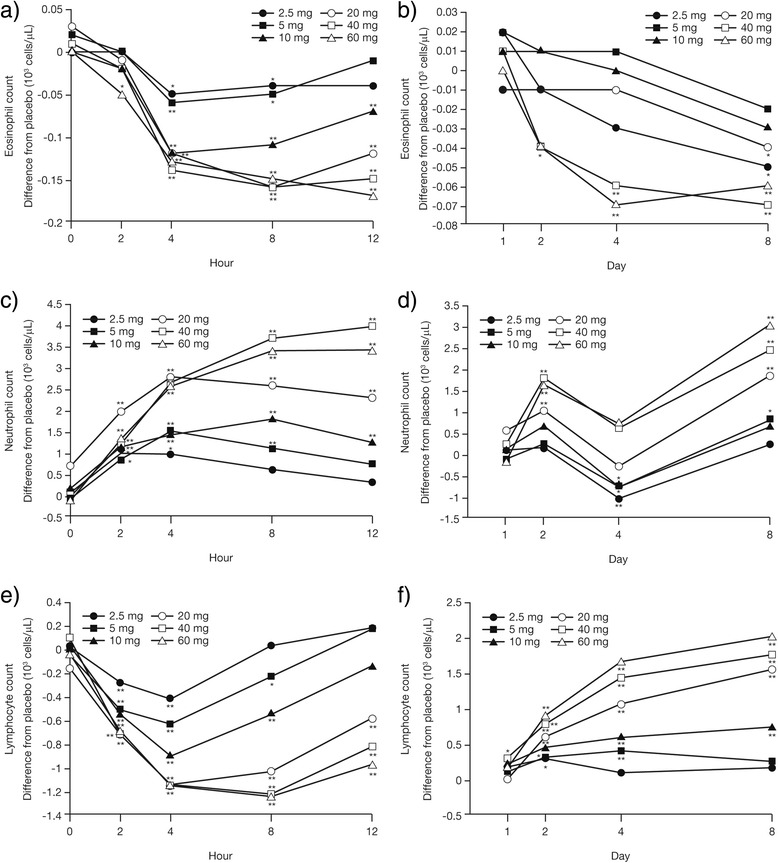
Mean change from baseline (difference from placebo) in white blood cell counts. Eosinophil, neutrophil, and lymphocyte counts for Day 1 by hour (**a**, **c**, **e**) and for Days 1 through 8 (**b**, **d**, **f**) for each daily prednisone dose. *P ≤ 0.05 and **P ≤ 0.01 versus placebo

Daily doses of prednisone up to 60 mg resulted in dose- and time-dependent effects on white blood cell counts. Eosinophil counts relative to placebo demonstrated acute dose-dependent reductions on Day 1. A significant reduction versus placebo was observed as early as 2 h post-dose with prednisone 60 mg (Fig. 2a). At 4 h reductions were significant at all doses, and from 4–12 h counts relative to placebo were relatively stable (Fig. 2a). Reductions in eosinophil counts relative to placebo were seen at most doses on Day 8 (Fig. 2b).

Prednisone induced increases in neutrophil counts relative to placebo throughout Day 1, with significant increases seen with doses ≥10 mg at 12 h post-dose (Fig. 2c). Differences in neutrophil counts relative to placebo were variable over the next 7 days: significant increases were observed with higher doses on Days 2 and 8, whereas decreases, which were significant with the lower doses, were seen on Day 4 (Fig. 2d).

As was observed with neutrophil counts, lymphocyte counts demonstrated acute dose-dependent reductions versus placebo on Day 1, with significant reductions observed with all doses as early as 2 h post-dose (Fig. 2e). Reductions in lymphocyte counts relative to placebo were greatest with most doses at 4 h post-dose, and were similar to placebo with the lower doses at 12 h post-dose (Fig. 2e). Lymphocyte counts relative to placebo continued to rise over the treatment period, and significant increases were seen with prednisone doses ≥10 mg on Day 8 (Fig. 2f).

#### Bone metabolism

**Fig. 3:**
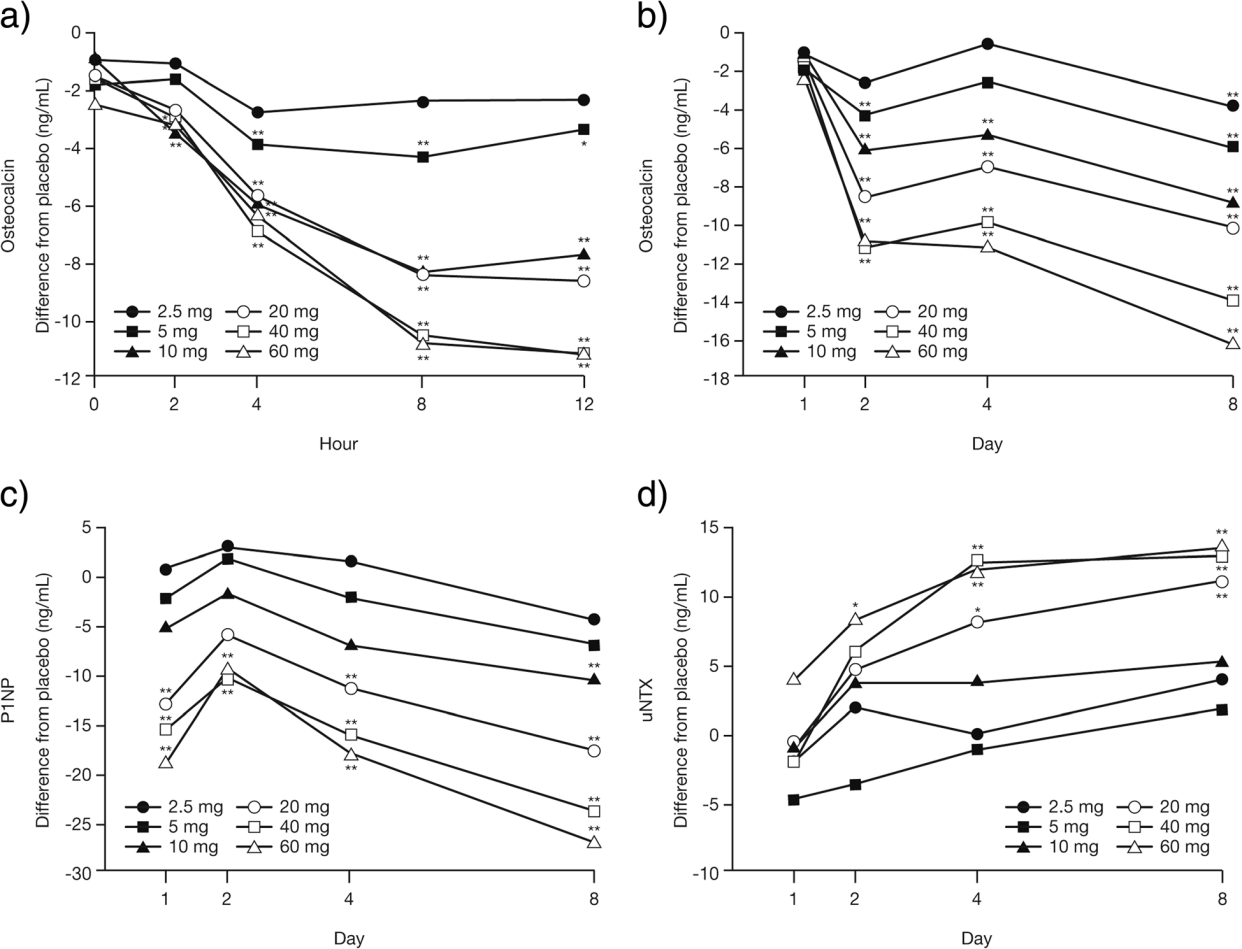
Mean change from baseline (difference from placebo) in biomarkers of bone metabolism. Change in osteocalcin (OC), procollagen type 1 N-propeptide (P1NP), and urinary N-terminal cross-linked telopeptide of type 1 collagen (uNTX) for each daily dose of prednisone. **a** OC: Day 1 by hour; (**b**) OC: Days 1–8; (**c**) P1NP: Days 1–8; (**d**) uNTX: Days 1–8. *P ≤ 0.05 and **P ≤ 0.01 versus placebo

Daily doses of prednisone up to 60 mg resulted in dose- and time-dependent effects on biomarkers of bone metabolism. OC and P1NP are biomarkers of bone formation. On Day 1, plasma OC significantly decreased relative to placebo as early as 2 h post-dose, and continued to decrease in a dose-dependent manner until 12 h post-dose (Fig. 3a). Reductions in plasma OC relative to placebo were significant for doses ≥5 mg on Day 2, and for all doses on Day 8 (Fig. 3b). P1NP was significantly reduced versus placebo with prednisone doses ≥20 mg on Day 1 (Fig. 3c). P1NP levels increased slightly relative to placebo on Day 2, and then decreased in a dose- and time-dependent manner until Day 8 (Fig. 3c).

Urinary NTX is a biomarker of bone loss. A significant increase in uNTX normalized for uCr (uNTX/uCr) versus placebo was observed with prednisone 60 mg by Day 2, and further dose-dependent increases occurred from Days 4–8 (Fig. 3d).

#### Metabolism

The results for fasting glucose and insulin on Days 1 and 8 are shown in Table 4. Differences in serum glucose concentrations between prednisone and placebo were small but significantly lower with prednisone 5 mg and doses ≥20 mg on Day 4, and doses ≥40 mg on Days 7 and 8. The effect of prednisone on glucose metabolism was tested using an OGTT on Day 6. Increases from baseline in both glucose and insulin concentrations at 0.5 h were observed for all prednisone doses and for placebo, followed by steady decreases for all prednisone doses at 1 and 2 h. After 6 days of prednisone treatment, the changes from baseline in both glucose and insulin concentrations relative to placebo were not significant at most time points (data not shown).

**Table 4:** Mean differences from placebo (standard error) in fasting glucose, fasting insulin, triglycerides, and adiponectin

Parameter	Day	Prednisone (mg/day)					
		2.5	5	10	20	40	60
Fasting glucose, mg/dL	1	1.19 (1.97)	3.44 (1.97)	1.60 (1.97)	0.94 (1.96)	1.86 (1.99)	2.13 (1.93)
	8	−2.06 (2.00)	−1.40 (2.00)	−2.11 (2.00)	−3.24 (1.99)	−6.66 (2.01)**	−7.10 (1.97)**
Fasting insulin, μU/mL	1	2.15 (2.90)	2.24 (2.81)	1.69 (2.90)	2.10 (2.89)	1.21 (2.83)	1.76 (2.90)
	8	−1.44 (2.34)	−1.70 (2.34)	−0.52 (2.34)	−0.39 (2.32)	−0.50 (2.34)	0.71 (2.31)
Triglycerides, mg/dL	1	39.19 (21.19)	17.93 (20.20)	22.60 (21.16)	85.79 (21.12)**	44.56 (20.52)*	37.18 (21.16)
	8	5.43 (17.01)	13.56 (16.95)	8.82 (17.01)	9.66 (16.95)	35.59 (17.09)*	26.42 (16.76)
Adiponectin, μg/mL	1	−1.51 (0.69)*	−1.61 (0.67)*	−0.47 (0.69)	−1.89 (0.69)*	−0.73 (0.68)	−0.18 (0.69)
	8	−0.27 (0.58)	−0.63 (0.58)	0.02 (0.58)	1.00 (0.58)	2.46 (0.58)**	2.47 (0.57)**

*P ≤ 0.05, **P ≤ 0.01 versus placebo

The effect of prednisone relative to placebo on serum triglyceride levels was variable. On Day 1, prednisone 20 mg and 40 mg significantly raised triglyceride levels. On Day 8, prednisone raised triglyceride levels, but the relationship to dose was inconsistent, and the impact generally was not significant (Table 4). Dose- and time-dependent effects of prednisone on adiponectin were also observed relative to placebo. On Day 8, adiponectin was significantly increased with higher prednisone doses (Table 4).

#### Central nervous system

The results of both the POMS™ and MOS-Sleep assessments did not show any changes when comparing the values to normative samples. However, no clear pattern for the treatment arms appeared in either assessment.

### Safety

There were no serious adverse events (SAEs) or deaths reported. There were no clinically significant changes in vital signs or body weight at any time point. The incidence of AEs with prednisone was not dose related. Treatment-emergent adverse events (TEAEs) were reported for two to eight subjects (13**–**50 %) across the prednisone doses and ten subjects (67 %) administered placebo. Central nervous system disorders, mainly headache, were the most frequently reported TEAEs (Table 5). Three subjects reported severe AEs; all were headaches experienced while on prednisone 2.5 mg, prednisone 20 mg, and placebo, respectively; the headache in the subject dosed with prednisone 2.5 mg was considered by the investigator to be related to the study treatment.

**Table 5:** Treatment-emergent adverse events (all events that occurred in >1 subject)

TEAE	TEAEs, *n* (%)						
		Prednisone (mg/day)					
	Placebo	2.5	5	10	20	40	60
	(*n* = 15)	(*n* = 15)	(*n* = 15)	(*n* = 15)	(*n* = 16)	(*n* = 15)	(*n* = 16)
Total	10 (67)	6 (40)	5 (33)	2 (13)	8 (50)	3 (20)	4 (25)
Headache	4 (27)	4 (27)	1 (7)	0	4 (25)	2 (13)	3 (19)
Nasopharyngitis	1 (7)	0	2 (13)	1 (7)	0	0	1 (6)
Pharyngolaryngeal pain	0	1 (7)	1 (7)	0	1 (6)	1 (7)	0
Cough	0	0	1 (7)	1 (7)	1 (6)	0	0
Dyshidrosis	1 (7)	0	0	0	1 (6)	1 (7)	0
Dyspepsia	0	0	1 (7)	0	1 (6)	1 (7)	0
Abdominal pain, upper	0	1 (7)	0	0	0	0	1 (6)
Dermatitis, contact	0	1 (7)	1 (7)	0	0	0	0
Dizziness	1 (7)	0	0	0	0	0	1 (6)
Dysmenorrhea	0	0	0	0	1 (6)	0	1 (6)
Furuncle	1 (7)	0	1 (7)	0	0	0	0
Nausea	0	0	0	0	0	1 (7)	1 (6)
Pyrexia	1 (7)	0	1 (7)	0	0	0	0

*TEAE* treatment-emergent adverse event

Treatment-related AEs were reported by two subjects (13 %) dosed with prednisone 2.5, 5, 20, and 40 mg, respectively, no subjects dosed with 10 mg, and three subjects (19 %) dosed with 60 mg. Treatment-related AEs were reported by four subjects (27 %) dosed with placebo. Headache was the most frequently reported treatment-related AE, occurring in one or two subjects (6 % or 13 %) across the prednisone doses and one subject dosed with placebo.

## Discussion

This study was designed to characterize the dose–response and time course of prednisone effects on biomarkers of GC receptor agonism in a healthy adult population over 7 days. Daily doses of prednisone up to 60 mg were generally well-tolerated and resulted in dose- and time-dependent effects on a number of biomarkers. As would be expected, a decrease relative to placebo was noted in biomarkers of bone formation (OC and P1NP), whereas there was an increase in a biomarker of bone turnover (uNTX). Also as expected, suppression of morning cortisol levels was seen at higher prednisone doses. Metabolic effects on glucose concentrations, OGTT, and triglyceride levels were modest and generally not statistically significant; however, adiponectin levels were significantly increased relative to placebo with higher prednisone doses by Day 8.

GCs are reasonably safe for short-term use. However, serious complications have often been reported with long-term use [10, 11]. In this study, inhibition of the HPA axis was evident by the potent, dose-dependent suppression of serum cortisol following the first dose of prednisone. As expected, with regard to the low-dose ACTH stimulation test, the subjects that took longer to return to normal were in the treatment groups that received the higher doses of prednisone in Period 3.

GCs are well known for their ability to affect circulating white blood cell profiles. It is generally acknowledged that administration of GC induces a transient fall in circulating lymphocytes, which is maximal 4–6 h after administration [19], particularly if the drug is administered in the morning [20]; this is thought to arise mainly from a reduced efflux of lymphocytes from lymphoid organs [13]. The transient fall is followed by a subsequent return to normal values within 12–24 h [19]. This was demonstrated in the present study, where all doses of prednisone reduced lymphocyte counts within 2 h, with maximum effect seen at 4–8 h. Levels started to return towards normal by 8 h after dosing, and were close to baseline values by 24 h. Repeated dosing with prednisone resulted in dose-dependent increases in lymphocyte counts, with the stimulation maintained for doses ≥10 mg on Day 8. The increases in lymphocyte count on the mornings prior to dosing were likely due to a rebound phenomenon reported previously for GCs [21]. Conversely, the dose-dependent decreases in eosinophils observed in the first 24 h continued through Day 8, albeit at a diminishing rate. The increased neutrophil counts observed following GC treatment are consistent with the literature, and are thought to be due to increased release from bone marrow and decreased movement out of the blood into tissue sites [21, 22].

Osteoporosis, a condition characterized mainly by a reduction in bone mineral density (BMD), is a well-established side effect of chronic GC therapy. In fact, chronic use of GCs increases the already increased risk of osteoporosis in patients with RA by twofold [23, 24]. Some studies suggest that the associated fractures actually occur at higher BMD levels in patients treated with GCs than in patients not treated with GCs [25]. However, low-dose GC therapy has been recognized to avert the deleterious effects of GCs seen at higher doses, possibly due its anti-inflammatory effect countering the bone loss caused by chronic inflammation; a literature review on the safety of long-term, low-dose GC therapy in patients with rheumatic diseases demonstrated that AEs can in fact be quite modest [26]. For example, the data from four extensively reviewed randomized controlled trials showed that BMD loss over 2 years of low-dose prednisone treatment is not significantly different from that with placebo. On the other hand, osteoporosis is still likely to be the most common side effect of chronic low-dose GC therapy [27].

Many studies maintain the idea that reduced bone formation is predominantly responsible for the GC-associated bone loss [28, 29]. OC, an osteoblast-derived protein involved in bone formation, is routinely utilized as a biomarker because of its close association with BMD [30]. In this study, plasma OC levels were significantly reduced as early as 2 h after the first administered prednisone doses above 10 mg on Day 1. This decrease was maintained throughout Day 1 and, consistent with the literature [31, 32], throughout the treatment period. A similar trend was also observed for P1NP, another biomarker of bone formation. Furthermore, prednisone increased uNTX, a biomarker of bone loss. Taken together, these data support both decreased bone formation and increased resorption, and demonstrate dose- and time-dependent effects of daily prednisone. Interestingly, biomarkers of bone turnover are thought to be useful in predicting the rate of bone loss in postmenopausal women [33]. In addition, some of these biomarkers, such as urinary C-telopeptide and free deoxypyridinoline, predict the associated threat of hip fracture independently of BMD [34], which is thought to be the most important predictor of osteoporotic fracture [35]. It has also been reported that several of these markers, such as serum OC and the CrossLaps peptide of urinary C-telopeptide, may be used to monitor the efficacy of therapy in patients with osteoporosis [36]. Furthermore, Garnero et al. reported that the rate of bone turnover plays an increasing role as a determinant of bone mass with increasing time following menopause, with high bone turnover being associated with low bone mass, and suggests that bone marker assessment may be useful in the evaluation of osteoporosis risk [37]. Thus, the bone biomarker data in the present study have potential predictive value for subsequent bone-related AEs of GCs.

This study carefully and thoroughly characterized the dose–response of prednisone on two significant safety concerns associated with use of GC: HPA axis suppression and adverse effects on bone metabolism. The dose- and time-dependent responses to prednisone on the HPA axis and bone biomarkers can be used for comparison with novel glucocorticoid receptor agonists. To demonstrate preliminary evidence of dissociation, however, it is essential to characterize dose–response for putative anti-inflammatory biomarkers of GCs. In healthy volunteers there is no ongoing inflammation that can be assessed for evidence of dose-dependent suppression. The effects on trafficking of circulating leukocytes may serve as a biomarker for anti-inflammatory effects in healthy volunteers. While not true anti-inflammatory biomarkers, they are likely to be associated with similar GC agonistic effects.

## Conclusions

This characterization of the dose–response of prednisone on various biomarkers of GC agonism provides important and relevant information on safety and PD responses associated with short-term prednisone dosing over the commonly used clinical dose range, and provides a reference for early clinical development of dissociated agents targeting a differentiated PD profile.

## Abbreviations

ACTH, adrenocorticotropic hormone; AE, adverse event; BMD, bone mineral density; DAGR, dissociated agonist of the glucocorticoid receptor; GC, glucocorticoid; HPA, hypothalamic-pituitary-adrenal; MOS-Sleep, medical outcomes study: sleep scale; OC, osteocalcin; OGTT, oral glucose tolerance test; P1NP, procollagen type 1 N-propeptide; PD, pharmacodynamics; POMS^TM^, profile of mood state; RA, rheumatoid arthritis; TEAE, treatment-emergent adverse event; uCR, urinary creatinine; uNTX, urinary N-terminal cross-linked telopeptide of type 1 collagen
